# Elevated TG/HDL-C and non-HDL-C/HDL-C ratios predict mortality in peritoneal dialysis patients

**DOI:** 10.1186/s12882-020-01993-5

**Published:** 2020-08-03

**Authors:** Wenkai Xia, Xiajuan Yao, Yan Chen, Jie Lin, Volker Vielhauer, Hong Hu

**Affiliations:** 1 Department of Nephrology, The Affiliated Jiangyin Hospital of Southeast University Medical College, 3 Yinrui Road, Jiangyin, 214400 Jiangsu China; 2 Nephrologisches Zentrum, Medizinische Klinik und Poliklinik IV, Klinikum der Universität München, Ludwig-Maximilians-University Munich, Munich, Germany

**Keywords:** Triglyceride, High-density lipoprotein cholesterol, Peritoneal dialysis, Prognosis

## Abstract

**Background and aims:**

Dyslipidemia is common in patients with chronic kidney disease and particular prevalent in patients receiving peritoneal dialysis. However, whether markers of atherogenic dyslipidemia correlate with outcomes in dialysis patients as in the general population is uncertain. The aim of this study was to explore the prognostic value of the serum triglyceride/HDL cholesterol (TG/HDL-C) ratio and non-HDL-C/HDL-C ratio to predict mortality in peritoneal dialysis patients.

**Methods:**

Two hundred fourteen peritoneal dialysis patients were retrospectively analyzed from January 2011 to December 2015, with a median follow-up of 59 months. We used receiver operating curves (ROC) to determine the optimal threshold for TG/HDL-C and non-HDL/HDL-C ratios at baseline to predict overall survival during follow-up. Prognostic values were accessed by univariate and multivariate COX regression analysis and Kaplan-Meier curve. A predictive nomogram was developed to predict prognosis for overall survival, and the predictive accuracy was evaluated by concordance index (c-index).

**Results:**

The optimal cut-off values for TG/HDL-C ratio and non-HDL-C/HDL-C ratio to predict mortality were 1.94 and 2.86, respectively. A high TG/HDL-C ratio and a high non-HDL-C/HDL-C ratio strongly correlated with worse overall survival in peritoneal dialysis patients. Multivariate analysis demonstrated that elevated TG/HDL-C ratio (HR 3.57, 95% CI 1.99, 6.39, *P* < 0.000) as well as non-HDL/HDL-C ratio (HR 2.58, 95%CI 1.39–4.81, *P* = 0.003) were independent markers to predict reduced OS. A nomogram was constructed to predict overall survival, with a c-index for predictive accuracy of 0.795.

**Conclusion:**

TG/HDL-C ratio and non-HDL-C/HDL-C may serve as potential prognostic biomarkers in PD patients.

## Background

The increasing prevalence of chronic kidney disease (CKD) is a worldwide public health issue. Despite dialysis treatment, patients with CKD still have an excessive risk for cardiovascular (CV) events, lower quality of life and high mortality [1]. Therefore, it is necessary to identify and better manage patients with risk factors for worse outcomes in CKD. Dyslipidemia is a common complication in CKD patients, especially among those receiving peritoneal dialysis (PD) treatment, which leads to high levels of triglyceride (TG) accompanied by low levels of high-density lipoprotein cholesterol (HDL-C) [2, 3]. The combination of high TG and low HDL-C has been identified as an independent predictor of cardiovascular disease (CVD) and all-cause mortality in non-CKD patients, with its ratio being of greater predictive value than the individual lipid measures alone [4–7]. An increased TG/HDL-C ratio predicted the development of nonfatal CVevents [7–9], CV death [10, 11], and all-cause mortality [12, 13] in healthy individuals and patients with increased CVD risk.

Several studies have reported that an elevated TG/HDL-C ratio correlated with the prevalence of CKD [14, 15]. However, conflicting data were reported on the association of high TG/HDL-C ratios with CVD and mortality in dialysis patients. Contrary to non-CKD patients, a large retrospective study in hemodialysis (HD) patients reported that high TG/HDL-C ratios were associated with reduced CV events and improved survival [16]. In contrast, Chen et al. demonstrated that a higher TG/HDL-C ratio was associated with increased CVD risk and mortality in prevalent dialysis patients including both HD and PD patients [17]. Indeed, further studies evaluating the prognostic utility of the TG/HDL-C ratio specifically in PD patients found that higher values were significantly associated with CVD mortality in female PD patients [18], and with higher all-cause and CVD mortality in older patients on PD. [12]

In addition, it has been proposed that the non-HDL-C/HDL-C ratio can be utilized as a simple indicator for CVD risk [19]. Non-HDL-C includes all the atherogenic lipoproteins and is calculated as total cholesterol (TC) minus HDL-C. However, data on the association of the non-HDL-C/HDL-C ratio with prognosis of patients on dialysis is limited.

In this study, we wanted to substantiate the positive association of higher TG/HDL-C ratios with overall survival (OS) in incident PD patients. Furthermore, we compared the prognostic impacts of TG/HDL-C and non-HDL-C/HDL-C ratios in PD patients and established prognostic nomograms to better predict outcomes in PD patients.

## Methods

### Patients

This was a single-center retrospective observational cohort study. Medical records of 243 incident PD patients were collected between January 2011 and December 2017 at the Affiliated Jiangyin Hospital of Southeast University Medical College. Exclusion criteria were as follows: patients aged < 18 years old and patients receiving less than 3 consecutive months of PD, a history of previous HD or renal transplantation, and patients lost to follow up. Finally, 214 patients were enrolled in this study. Baseline characteristics at the first 1–3 months after the initiation of CAPD were collected. The primary endpoint was all-cause mortality. Each patient was followed up until death or censoring on December 31th, 2017. All patients were regularly followed-up with physical examination, and laboratory testing. Informed consent was waived by the Ethical Committee due to the retrospective and non-interventional nature of the study.

### Analysis of blood samples

Peripheral blood was obtained for the measurement of laboratory values, including hemoglobin, albumin, serum creatine, blood urea nitrogen, uric acid, calcium, phosphorus, potassium, serum triglyceride, total cholesterol, HDL-C and LDL-C. Intact parathyroid hormone (iPTH) level was measured by immunoassay.

### Definition and optimal cutoff values of TG/HDL-C ratio and non-HDL/HDL-C ratio

TG/HDL-C ratio was defined as serum levels of triglyceride (TG) divided by high-density lipoprotein cholesterol (HDL-C). Non-HDL-C was HDL-C subtracted by total cholesterol (TC), and non-HDL/HDL-C ratio was defined as non-HDL-C divided by HDL-C. Variance inflation factor was used to measure multicollinearity, with a variance inflation factor ≥ 10 indicating collinearity. Receiver operating curve (ROC) analyses was applied to determine the optimal cut-off value of TG/HDL-C ratio and non-HDL/HDL-C ratio. Using OS as endpoint, optimal thresholds of TG/HDL-C ratio and non-HDL/HDL-C ratio were obtained according to the highest Youden’s index. Subsequently, patients were divided into two groups based on the optimal thresholds.

### Statistical analysis

Comparison of categorical variables was conducted by the Pearson X^2^ test. Comparison of continuous variables was analyzed with Mann-Whitney U or Kruskal-Wallis test. Survival rates were evaluated through the Kaplan-Meier method with log-rank test. R 3.0.3 software with the package *rms* was used to establish the nomogram and calibration curve. The predictive accuracy was evaluated using Harrell’s concordance index (c-index). The Cox proportional hazards regression model was performed in univariate analysis and the significant variables of univariate analysis were calculated into the multivariable analysis. All statistical analysis was performed by SPSS 20.0 software (SPSS Inc., IBM, USA) and R software version 3.2.2 (Institute for Statistics and Mathematics, Vienna, Austria).

## Results

### Baseline characteristics

A total of 214 incident PD patients were finally enrolled in this study. The clinical and biochemical baseline characteristics of all PD patients according to low versus high serum TG/HDL-C ratio and non-HDL/HDL-C ratio are summarized in Table 1. The mean age of patients was 50 ± 14 years, 59% were men. The median follow-up period was 59 months ranging from 3 to 60 months. Fifty-four patients died from any cause during the follow-up period. The median value of TG/HDL-C ratio and non-HDL/HDL-C ratio was 1.33 (range 0.16–9.47) and 2.88 (range 0.56–10.94), respectively.

**Table 1:** Baseline characteristics according to TG/HDL-C ratio and non-HDL/HDL-C ratio

Variable	Cases (*n* = 214)	TG/HDL-C ratio		*P*	Non-HDL/HDL-C ratio		*P*
		TG/HDL-c < 1.94 *n* = 152	TG/HDL-c ≥ 1.94 *n* = 62		Non-HDL/HDL-C < 2.86 *n* = 109	Non-HDL/HDL-C ≥ 2.86 *n* = 105	
Age, y		49 ± 14	53 ± 13	0.021	49 ± 15	51 ± 14	0.393
Male, (n, %)		88 (57.9)	38 (61.3)	0.647	69 (63.3)	57 (54.3)	0.180
BMI		21.8 ± 2.6	23.1 ± 3.1	0.476	22.1 ± 2.7	23.2 ± 2.9	0.519
Diabetes (%)		25 (16.4)	16 (25.8)	0.115	17 (15.6)	24 (22.9)	0.177
CVD (%)		10 (6.6)	5 (9.6)	0.699	8 (7.3)	7 (6.7)	0.847
Hypertension (%)		79 (52.0)	43 (69.4)	0.020	58 (53.2)	64 (61.0)	0.253
Systolic pressure (mmHg)		149.0 ± 22.2	146.2 ± 22.4	0.871	149.8 ± 22.0	146.5 ± 22.5	0.932
Diastolic pressure (mmHg)		90.4 ± 15.2	87.8 ± 15.1	0.322	90.6 ± 14.7	88.6 ± 15.7	0.833
Total Kt/V		2.08 (1.57, 2.68)	2.06 (1.54, 2.63)	0.183	2.09 (1.65,2.71)	2.11 (1.73, 2.74)	0.956
Laboratory data							
Hemoglobin, g/dL		97.9 (86.3, 108.0)	98.5 (85.5, 109.2)	0.772	97.7 (84.8, 108.0)	99.0 (87.4, 109.5)	0.619
Albumin, g/L		34.5 ± 4.1	34.3 ± 4.3	0.733	34.6 ± 4.2	34.3 ± 4.1	0.580
Creatinine, umol/L		813.6 (631.8, 1091.1)	847.6 (738.2, 1047.1)	0.822	871.9 (660.8, 1100.0)	825.3 (683.0, 1026.2)	0.681
BUN, mmol/L		17.2 (13.5, 20.9)	16.9 (13.9, 20.4)	0.569	17.2 (14.3, 21.3)	16.9 (13.4, 20.2)	0.383
Uric acid, umol/L		430.6 (378.2, 489.6)	463.8 (392.8, 546.0)	0.036	426.0 (379.8, 489.3)	451.6 (371.7, 517.0)	0.117
K, mmol/L		3.9 (3.3, 4.5)	3.7 (3.3, 4.2)	0.197	3.9 (3.3, 4.5)	3.8 (3.3, 4.4)	0.900
Ca, mmol/L		2.1 (2.0, 2.2)	2.1 (1.9, 2.3)	0.755	2.0 (1.9, 2.2)	2.1 (2.0, 2.4)	0.047
Na, mmol/L		140.0 (136.3, 142.6)	139.0 (134.9, 142.3)	0.086	139.7 (135.9, 142.3)	140.0 (137.0, 142.9)	0.486
P, mmol/L		1.5 (1.2, 1.9)	1.7 (1.4, 2.0)	0.302	1.6 (1.3, 1.9)	1.6 (1.2, 2.0)	0.631
iPTH, pmol/L		24.0 (11.9, 64.2)	24.9 (10.4, 52.9)	0.805	23.0 (13.9, 68.5)	25.0 (7.5, 48.5)	0.966
TC, mmol/L		1.3 ± 0.6	4.6 ± 1.3	0.770	4.1 ± 1.0	5.0 ± 1.4	< 0.001
TG, mmol/L		1.2 (0.9, 1.5)	2.6 (2.2, 3.3)	0.000	1.2 (0.8, 1.5)	2.0 (1.4, 3.0)	< 0.001
HDL-C, mmol/L		1.2 (1.0, 1.5)	0.9 (0.8, 1.0)	< 0.001	1.3 (1.1, 1.6)	1.0 (0.8, 1.2)	< 0.001
LDL-C, mmol/L		1.8 (1.3,2.5)	1.8 (1.1, 2.3)	0.158	1.6 (1.2, 2.2)	2.0 (1.5, 2.7)	0.002
Non-HDL-C, mmol/L		3.2 ± 1.2	3.6 ± 1.2	0.014	2.7 ± 0.8	4.0 ± 1.2	< 0.001
Medications							
Statin/Fibrate, n (%)		48 (31.6)	39 (62.9)	< 0.001	31 (28.4)	77 (73.3)	< 0.001

Values are expressed as mean ± SD, median and interquartile range, or number (percentage) as appropriate. *CVD* Cardiovascular disease, *TG* triglyceride, *TC* total cholesterol, *BUN* blood urea nitrogen, *HDL-C* high-density lipoprotein cholesterol, *LDL-C* low-density lipoprotein cholesterol, *iPTH* intact parathyroid hormone

### The optimal cutoff value for TG, HDL-C, TG/HDL-C and non-HDL/HDL-C

**Fig. 1:**
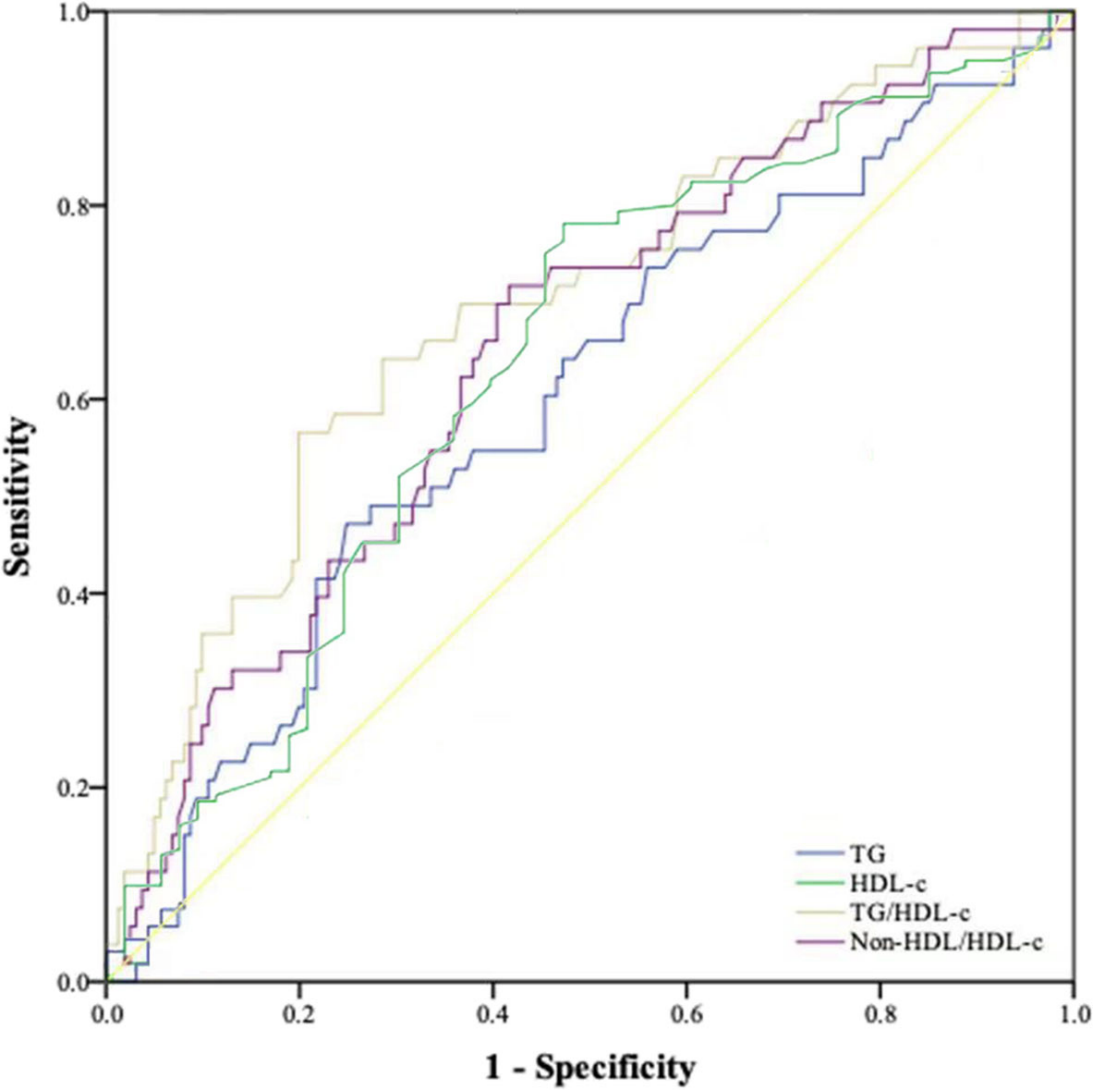
Optimal cutoff value for TG, HDL-C, TG/HDL-C ratio and non-TG/HDL-C ratio were applied with ROC curves for survival status

The optimal thresholds of TG, HDL, TG/HDL-C and non-HDL/HDL-C were determined using receiver operating curve (ROC) analysis (Fig. 1). The optimal cutoff levels of TG, HDL-C, TG/HDL-C and non-HDL/HDL-C based on the highest Youden’s index were 1.47 mmol/L (AUC: 0.598, 95%CI: 0.509–0.687, *P* = 0.032), 0.99 mmol/L (AUC: 0.637, 95%CI: 0.274–0.451, *P* = 0.003), 1.94 (AUC: 0.696, 95%CI: 0.612–0.779, *P* = 0.000), and 2.86 (AUC: 0.653, 95%CI: 0.570–0.737, *P* = 0.003), respectively.

### Associations of TG/HDL-C and non-HDL/HDL-C with patients’ outcomes

**Fig. 2:**
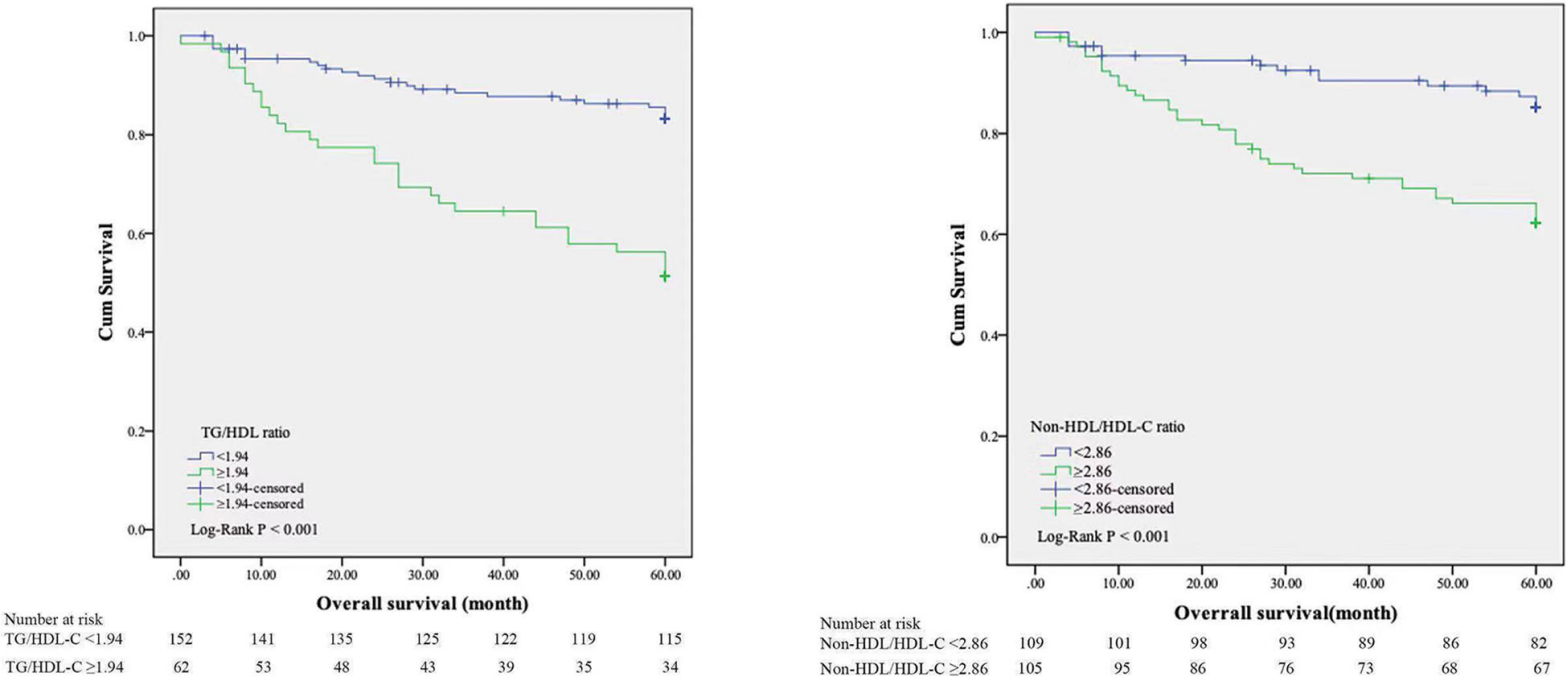
Kaplan-Meier curves for overall survival according to TG/HDL-C ratio and non-HDL-C/HDL-C ratio

Kaplan-Meier survival analysis and log-rank tests were used to determine the association of TG/HDL-C and non-HDL/HDL-C with patients’ survival. Our results demonstrate that TG/HDL-C ≥ 1.94 and non-HDL/HDL-C ≥ 2.86 were significantly associated with decreased OS (Fig. 2, *P* < 0.001). Furthermore, results from multivariate Cox regression analysis revealed that an elevated TG/HDL-C ratio was independently associated with reduced OS (HR 3.57, 95% CI 1.99–6.39, *P* < 0.001). Patients with non-HDL/HDL-C ≥ 2.86 had also an increased risk for all-cause mortality compared to patients with non-HDL/HDL-C<2.86 (HR 2.58, 95%CI 1.39–4.81, *P* = 0.003). In addition, age (HR 1.04, 95%CI 1.01–1.07, *P* = 0.005), iPTH (HR 0.99, 95%CI 0.99–1.00, *P* = 0.021), TG (HR 1.36, 95%CI 1.17–1.58, *P* < 0.001) and HDL-C (HR 0.27, 95%CI 0.11–0.68, *P* = 0.005) were independent indictors for OS of PD patients (Table 2).

**Table 2:** Univariate and multivariate analysis of prognostic factors of overall survival by Cox regression model

Variable	Overall survival			
	Univariate analysis		Multivariate analysis	
	HR (95% CI)	*P* value	HR (95% CI)	*P* value
Age	1.05 (1.03–1.07)	**< 0.001**	1.04 (1.01–1.07)	**0.005**
Gender (male)	0.83 (0.48–1.41)	0.483		
BMI	1.34 (0.79–2.27)	0.286		
Diabetes	2.13 (1.19–3.78)	**0.010**	1.08 (0.56–2.10)	0.809
CVD	2.80 (1.32–5.94)	**0.007**	0.87 (0.34–2.21)	0.867
Hypertension	2.35 (1.28–4.31)	**0.006**	1.10 (0.54–2.22)	0.795
Systolic pressure	1.00 (0.99–1.01)	0.862		
Diastolic pressure	0.98 (0.96–1.00)	0.080		
Total Kt/V	1.16 (0.69–1.96)	0.579		
Hemoglobin	1.00 (0.99–1.01)	0.805		
Albumin	0.95 (0.89–1.02)	0.135		
Creatinine	1.05 (0.95–1.16)	**0.003**	1.00 (0.99–1.00)	0.178
BUN	0.97 (0.93–1.02)	0.218		
Uric acid	1.00 (1.00–1.00)	0.642		
K	1.01 (1.00–1.02)	0.163		
Ca	1.31 (0.55–3.11)	0.547		
Na	0.99 (0.98–1.00)	0.251		
P	0.86 (0.59–1.25)	0.430		
iPTH	0.99 (0.99–1.00)	**0.022**	0.99 (0.99–1.00)	**0.021**
TC	1.21 (0.99–1.48)	0.066		
TG	1.37 (1.21–1.56)	**< 0.001**	1.36 (1.17–1.58)	**< 0.001**
HDL-C	0.42 (0.19–0.92)	**0.030**	0.27 (0.11–0.68)	**0.005**
LDL-C	0.90 (0.71–1.40)	0.990		
Non-HDL-C	1.35 (1.10–1.66)	**0.005**	1.23 (0.97–1.55)	0.083
TG/HDL-C	3.56 (2.08–6.10)	**< 0.001**	3.57 (1.99–6.39)	**< 0.001**
Non-HDL/HDL-C	2.99 (1.65–5.44)	**< 0.001**	2.58 (1.39–4.81)	**0.003**
Therapy of Statin/Fibrate	0.89 (0.72–1.21)	0.079		

*CVD* Cardiovascular disease, *TG* triglyceride, *TC* total cholesterol, *BUN* blood urea nitrogen, *HDL-C* high-density lipoprotein cholesterol, *LDL-C* low-density lipoprotein cholesterol; iPTH, intact parathyroid hormone

### New prognostic model for OS

**Fig. 3:**
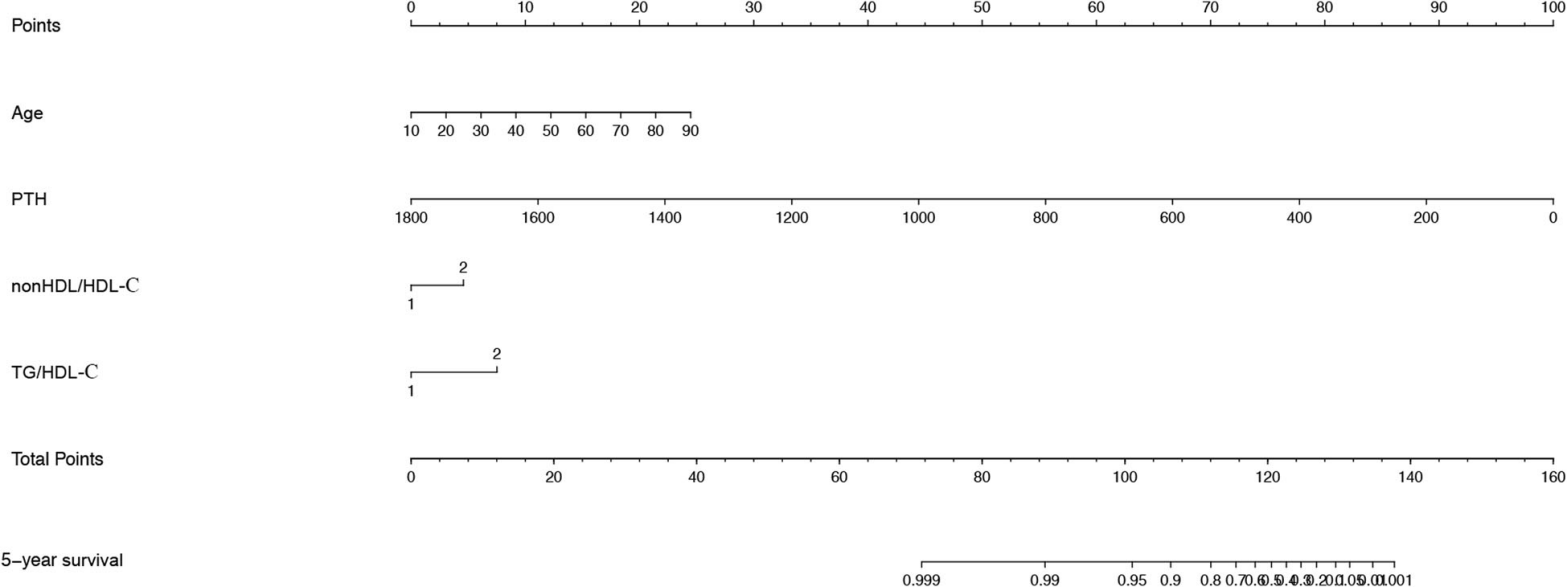
Nomogram for predicting 5-year survival of peritoneal dialysis patients

**Fig. 4:**
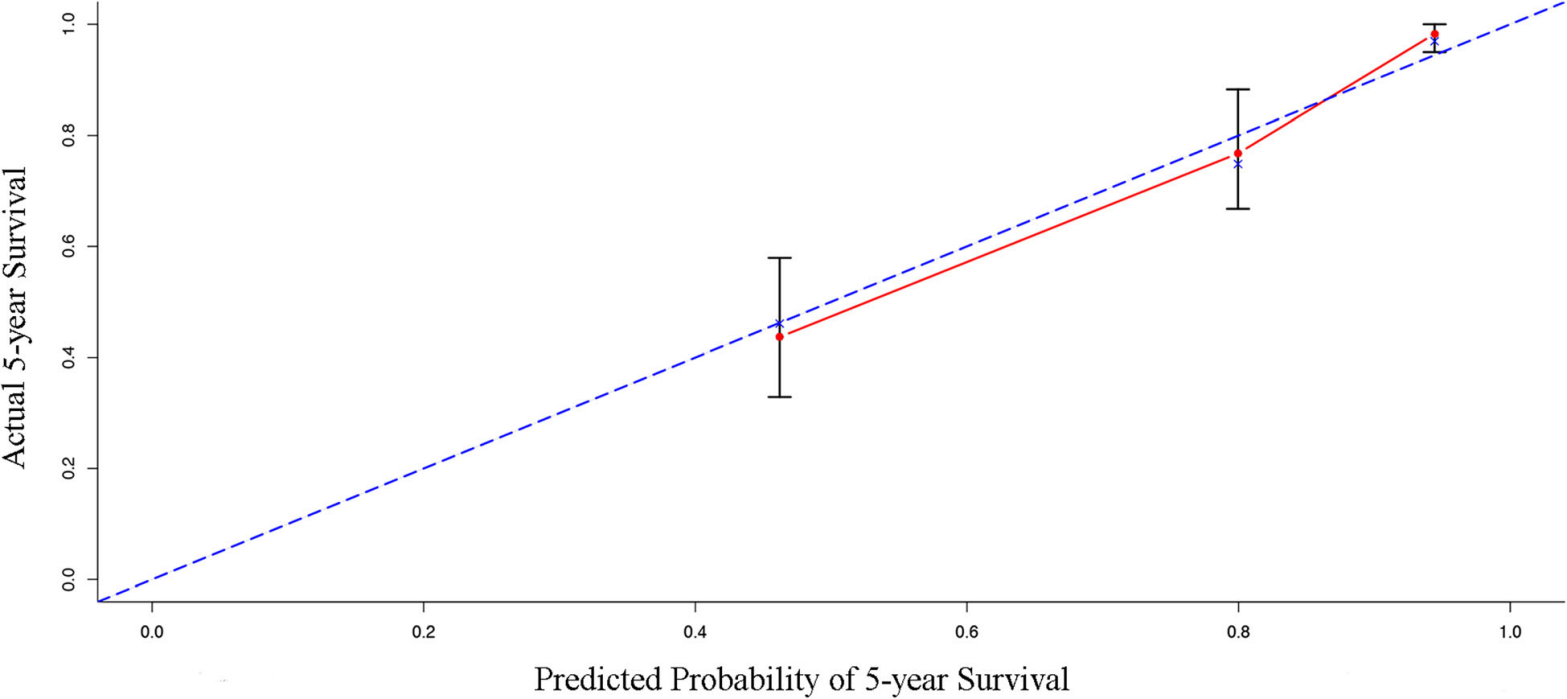
Calibration plot of the nomogram for 5-year overall survival. Notes: The 45-degree reference line represents the performance of a perfect nomogram. The red dashed line shows the performance of the observed nomogram. It seems that the nomogram precisely predicts the 5-year OS. *n* = 190; d = 50; *P* = 4.5;50 subjects per group; X-resampling optimism added, B = 200; comparison between nomogram-predicted probability of OS (X-axis) and the actual 5-year survival (Y-axis)

To predict survival of PD patients, we developed a nomogram by integrating all the independent prognostic factors according to the results from the Cox regression model (Fig. 3). The nomogram demonstrated that old age, a high TG/HDL-C and a high non-HDL/HDL-C ratio were indictors of a poor prognosis, whereas a high iPTH was a favorable factor. These results were similar to those obtained in the multivariate analyses (Table 2). To access the predictive accuracy of the nomogram, we calculated the c-index of the nomogram for OS prediction, which was 0.795. The performance of the nomogram to predict 5-year OS was verified by calibration plots (Fig. 4).

## Discussion

In this retrospective cohort study, we evaluated the prognostic performance of TG/HDL-C and non-HDL/HDL-C ratios in PD patients to predict OS. An elevated serum TG/HDL-C ratio was most significantly associated with higher all-cause mortality, but also the non-HDL/HDL-C ratio could be identified as an indicator for OS in PD patients. In addition, we developed a novel nomogram incorporating these ratios to improve predictive accuracy for mortality in PD patients.

Dyslipidemia is an important CVD risk factor in the general population and is prevalent in CKD and PD patients [2, 3]. However, in contrast to non-CKD patients serum LDL-C levels have not been identified as a strong risk factor for CVD in end stage renal disease patients undergoing dialysis [13, 20]. Consistently, statin therapy to lower LDL-C did not lead to reduced CVD and mortality in HD patients in respective clinical trials [21–23]. Independent of LDL-C levels, elevated serum TG and reduced HDL-C have been identified as risk factors for CVD, and the combination of these measures as a TG/HDL-C ratio was found to better predict risk for CVD and mortality than individual markers alone [5–7]. In CKD, impaired clearance of very low-density lipoproteins (VLDL) and chylomicrons lead to hypertriglyceridemia and deficiency of HDL, with defective HDL antioxidant, anti-inflammatory, and reverse cholesterol transport (RCT) activity [24]. Consistently, higher TG/HDL-C ratios were associated with the presence of CKD in cross-sectional studies [14, 15], and predicted the development of CKD in patients with type 2 diabetes [25]. Prior studies also found a significant association between higher TG/HDL-C ratios and progression of diabetic kidney disease or the risk for CVD events after renal transplantation [26, 27].

Therefore, several studies investigated whether an elevated TG/HDL-C ratio was also a risk factor for the development of CVD and mortality in the dialysis population but yielded conflicting results. Indeed, a large retrospective study in incident HD patients demonstrated that a higher TG/HDL-C ratio correlated with reduced CVD and better OS [16], indicating a complex and even paradoxical relationship of dyslipidemia and clinical risk in this patient population. In contrast, studies enrolling in part or exclusively PD patients reported a positive association of an increased TG/HDL-C ratio with CVD risk and mortality, particularly in female and older patients [12, 17]. Importantly, the results of our study confirm the independent relationship between a high TG/HDL-C ratio and OS in PD patients. We found that an increased TG/HDL-C ratio was independently correlated with all-cause mortality in PD patients, and the optimal threshold of 1.94 for the TG/HDL-C ratio was the best predictor in terms of hazard ratio (HR), and achieved the highest specificity and sensitivity. However, the applicable cut-off value was different from other studies, which may be due to differences in geographic region and race [11]. Reasons for the opposing relationship of the TG/HDL-C ratio with CVD risk and mortality in PD versus HD patients are not clear. Interestingly, the TG/HDL-C ratio is also a predictor for insulin resistance [28–33], which may be particularly prevalent in PD patients and is associated with an increased risk of hyperglycemia, dyslipidemia, and hypertension, all of which drive CVD mortality.

Our study extends previous findings by additionally identifying the non-HDL-C/HDL-C ratio as a positive predictor for OS in PD patients. Non-HDL-C/HDL-C may correlate better with CVD risk than LDL-C and non-HLD-C levels [34]. Similar to the TG/HDL-C ratio associations of high non-HDL-C/HDL-C ratios were reported with CVD in the general population [19, 35], with insulin resistance [36], and with CKD in an adult Chinese population [15]. Furthermore, we found low level of iPTH was associated with poor prognosis, which was consistent with Avram’s study [37]. This may be because elevated iPTH is a surrogate for higher caloric and protein intake, consequently leading to improved survival on PD dialysis. To the best of our knowledge, this is the first study to demonstrate that the non-HDL/HDL-C ratio is a potential prognostic marker for OS in PD patients. In the current study, a non-HDL/HDL-C ratio ≥ 2.84 was an independent indicator of overall mortality in PD patients after 5 years of follow-up.

However, based on hazard ratios the TG/HDL-C ratio out-performed non-HDL/HDL-C in predicting OS. Further studies are needed to test whether the non-HDL/HDL-C ratio can predict CV outcomes in CKD patients.

As reported previously [4–7, 38], elevated TG/HDL-C and non-HDL-C/HDL-C ratios better predicted OS in this study population than levels of TG, HDL-C and non-HDL-C alone. Indeed, despite the known protective cardiovascular functions of HDL-C, including reverse cholesterol transport, antioxidant, anti-inflammatory and anti-thrombotic properties [24], high HDL-C levels did not associate with all-cause mortality in patients with reduced kidney function in a large cohort study [39]. These observations were supported by studies investigating HDL-C cholesterol efflux capacity (CEC) as a marker of HDL-C functionality, in which CEC did not predict CV events or mortality in dialysis patients [40, 41]. Although high TG and low HDL-C levels were independently associated with mortality in our cohort after multivariate analysis, LDL-C and non-HDL-C were not, despite the predictive value of a higher non-HDL-C/HDL-C ratio. Together, these results suggest that in CKD and PD patients the TG/HDL-C and non-HDL-C/HDL-C ratios better reflect the balance between pro-atherogenic and protective lipoproteins affecting relevant patient outcomes, i.e. CVD and mortality.

The nomogram is a visual and generally accepted way to predict overall survival utilizing multiple clinical characteristics [42]. Several nomograms have been used to predict disease prognosis based on clinical characteristics, and nomograms were considered to be more precise than a traditional staging system for predicting prognosis in tumors [43]. However, few studies have demonstrated whether nomograms can predict outcomes in PD patients. The current study established a prognostic nomogram to predict 5-year mortality in PD patients including the TG/HDL-C and non-HDL-C/HDL-C ratios which we identified as independent prognostic markers. The nomogram performed well for OS, which was supported by the obtained c-index (0.795). Our results demonstrated that the derived nomogram could be a valuable tool to predict prognosis in patients undergoing PD.

This study has several limitations. First, this was a retrospective study based on a single-center database with a relatively small number of 214 patients, which may have resulted in bias for data collection and analysis. Second, other factors such as insulin resistance, mean peritoneal glucose load and smoking, which may be associated with mortality in PD patients, are not mentioned in our study. In addition, no data are available on the relationship between TG/HDL-C or non-HDL/HDL-C ratios with CVD mortality, a sub-analysis on the of TG/HDL-C and non-HDL/HDL-C ratios as predictors of CV events and CV mortality should be considered in the future analysis.

## Conclusion

In conclusion, our study demonstrated that TG/HDL-C ratio and non-HDL/HDL-C ratio were independent predictors of OS in PD patients. TG/HDL-C ratio was a better predictor of OS than non-HDL/HDL-C ratio. TG/HDL-C and non-HDL/HDL-C ratios and the newly developed predictive nomogram may be valuable to determine the clinical prognosis and may help to establish optimal therapeutic strategies.

## Data Availability

The datasets used and/or analyzed during the current study are available from the corresponding author on reasonable request.

## Abbreviations

CKD: Chronic kidney disease
PD: Peritoneal dialysis
TG: Triglyceride
HDL-C: High-density lipoprotein cholesterol
CVD: Cardiovascular disease
HD: Hemodialysis
TC: Cholesterol
OS: Overall survival
iPTH: Intact parathyroid hormone
ROC: Receiver operating curve
LDL-C: Low-density lipoprotein cholesterol
BUN: Blood urea nitrogen
VLDL: Very low-density lipoproteins
RCT: Reverse cholesterol transport
HR: Hazard ratio

## References

[1] Slinin Y, Greer N, Ishani A (2015). Timing of dialysis initiation, duration and frequency of hemodialysis sessions, and membrane flux: a systematic review for a KDOQI clinical practice guideline. Am J Kidney Dis.

[2] Ferro CJ, Mark PB, Kanbay M (2018). Lipid management in patients with chronic kidney disease. Nat Rev Nephrol.

[3] Park CH, Kang EW, Park JT (2017). Association of serum lipid levels over time with survival in incident peritoneal dialysis patients. J Clin Lipidol.

[4] Sultani R, Tong DC, Peverelle M (2020). Elevated triglycerides to high-density lipoprotein cholesterol (TG/HDL-C) ratio predicts long-term mortality in high-risk patients. Heart Lung Circ.

[5] Gaziano JM, Hennekens CH, O’Donnell CJ (1997). Fasting triglycerides, high-density lipoprotein, and risk of myocardial infarction. Circulation..

[6] Jeppesen J, Hein HO, Suadicani P (2001). Low triglycerides high high-density lipoprotein cholesterol and risk of ischemic heart disease. Arch Intern Med.

[7] Park JH, Lee J, Ovbiagele B (2014). Nontraditional serum lipid variables and recurrent stroke risk. Stroke..

[8] Barzi F, Patel A, Woodward M (2005). Asia Pacific cohort studies collaboration: a comparison of lipid variables as predictors of cardiovascular disease in the Asia Pacific region. Ann Epidemiol.

[9] Vega GL, Barlow CE, Grundy SM (2014). Triglyceride-to-high-density-lipoprotein-cholesterol ratio is an index of heart disease mortality and of incidence of type 2 diabetes mellitus in men. J Investig Med.

[10] Bittner V, Johnson BD, Zineh I (2009). The triglyceride/high-density lipoprotein cholesterol ratio predicts all-cause mortality in women with suspected myocardial ischemia: a report from the Women’s ischemia syndrome evaluation (WISE). Am Heart J.

[11] Wan K, Zhao J, Huang H (2015). The association between triglyceride/high-density lipoprotein cholesterol ratio and all-cause mortality in acute coronary syndrome after coronary revascularization. PLoS One.

[12] Zhan X, Yang M, Zhou R (2019). Triglyceride to high-density lipoprotein cholesterol ratio is associated with increased mortality in older patients on peritoneal dialysis. Lipids Health Dis.

[13] Tonelli M, Muntner P, Lloyd A (2013). Alberta kidney disease network: association between LDL-C and risk of myocardial infarction in CKD. J Am Soc Nephrol.

[14] Ho CI, Chen JY, Chen SY (2015). Relationship between TG/HDL-C ratio and metabolic syndrome risk factors with chronic kidney disease in healthy adult population. Clin Nutr.

[15] Wen J, Chen Y, Huang Y (2017). Association of the TG/HDL-C and non-HDL-C/HDL-C ratios with chronic kidney disease in an adult Chinese population. Kidney Blood Press Res.

[16] Chang TI, Streja E, Soohoo M (2017). Association of Serum Triglyceride to HDL cholesterol ratio with all-cause and cardiovascular mortality in incident hemodialysis patients. Clin J Am Soc Nephrol.

[17] Chen HY, Tsai WC, Chiu YL (2015). Triglyceride to high-density lipoprotein cholesterol ratio predicts cardiovascular outcomes in prevalent dialysis patients. Medicine (Baltimore).

[18] Hu H, Xiong L, Xu Q (2015). Higher serum triglyceride to high-density lipoprotein cholesterol ratio was associated with increased cardiovascular mortality in female patients on peritoneal dialysis. Nutr Metab Cardiovasc Dis.

[19] Qin G, Tu J, Zhang C (2015). The value of the apoB/apoAI ratio and the non-HDL-C/HDL-C ratio in predicting carotid atherosclerosis among Chinese individuals with metabolic syndrome: a cross-sectional study. Lipids Health Dis.

[20] Wanner C, Tonelli M (2014). Kidney disease: improving global outcomes lipid guideline development work group members: KDIGO clinical practice guideline for lipidmanagement in CKD: summary of recommendation statements and clinical approach to the patient. Kidney Int.

[21] Wanner C, Krane V, Marz W (2005). German diabetes and dialysis study investigators: atorvastatin in patients with type 2 diabetes mellitus undergoing hemodialysis. N Engl J Med.

[22] Fellstrom BC, Jardine AG, Schmieder RE (2009). AURORA study group: Rosuvastatin and cardiovascular events in patients undergoing hemodialysis. N Engl J Med.

[23] Baigent C, Landray MJ, Reith C (2011). SHARP investigators: the effects of lowering LDL cholesterol with simvastatin plus ezetimibe in patients with chronic kidney disease (study of heart and renal protection): a randomised placebo-controlled trial. Lancet..

[24] Vaziri ND (2014). Role of dyslipidemia in impairment of energy metabolism, oxidative stress, inflammation and cardiovascular disease in chronic kidney disease. Clin Exp Nephrol.

[25] Zoppini G, Negri C, Stoico V (2012). Triglyceride-high-density lipoprotein cholesterol is associated with microvascular complications in type 2 diabetes mellitus. Metabolism..

[26] Kim JE, Yu MY, Kim YC (2019). Ratio of triglyceride to high-density lipoprotein cholesterol and risk of major cardiovascular events in kidney transplant recipients. Clin Exp Nephrol.

[27] Yun KJ, Kim HJ, Kim MK (2016). Risk factors for the development and progression of diabetic kidney disease in patients with type 2 diabetes mellitus and advanced diabetic retinopathy. Diabetes Metab J.

[28] Iwani NA, Jalaludin MY, Zin RM (2017). Triglyceride to HDL-C ratio is associated with insulin resistance in overweight and obese children. Sci Rep.

[29] Uruska A, Zozulinska-Ziolkiewicz D, Niedzwiecki P (2018). TG/HDL-C ratio and visceral adiposity index may be useful in assessment of insulin resistance in adults with type 1 diabetes in clinical practice. J Clin Lipidol.

[30] Kannel WB, Vasan RS, Keyes MJ (2008). Usefulness of the triglyceride–high-density lipoprotein versus the cholesterol–high-density lipoprotein ratio for predicting insulin resistance and cardiometabolic risk (from the Framingham offspring cohort). Am J Cardiol.

[31] Kim JS, Kang HT, Shim JY (2012). The association between the triglyceride to high-density lipoprotein cholesterol ratio with insulin resistance (HOMA-IR) in the general Korean population: based on the National Health and nutrition examination survey in 2007-2009. Diabetes Res Clin Pract.

[32] Ren X, Chen ZA, Zheng S (2016). Association between triglyceride to HDL-C ratio (TG/HDL-C) and insulin resistance in Chinese patients with newly diagnosed type 2 diabetes mellitus. PLoS One.

[33] Young KA, Maturu A, Lorenzo C (2019). The triglyceride to high-density lipoprotein cholesterol (TG/HDL-C) ratio as a predictor of insulin resistance, beta-cell function, and diabetes in Hispanics and African Americans. J Diabetes Complicat.

[34] Lamprea-Montealegre JA, Sharrett AR, Matsushita K (2014). Chronic kidney disease, lipids and apolipoproteins, and coronary heart disease: the ARIC study. Atherosclerosis..

[35] Masson W, Epstein T, Huerín M (2019). Association between non-HDL-C/HDL-C ratio and carotid atherosclerosis in postmenopausal middle-aged women. Climacteric..

[36] Kim SW, Jee JH, Kim HJ (2013). Non-HDL-cholesterol/HDL-cholesterol is a better predictor of metabolic syndrome and insulin resistance than apolipoprotein B/apolipoprotein A1. Int J Cardiol.

[37] Morrell MA, Rajanna S, David KA (1996). Enrollment parathyroid hormone level is a new marker of survival in hemodialysis and peritoneal dialysis therapy for uremia. Am J Kidney Dis.

[38] Dai D, Chen B, Wang B (2016). Pretreatment TG/HDL-C ratio is superior to triacylglycerol level as an independent prognostic factor for the survival of triple negative breast cancer patients. J Cancer.

[39] Zewinger S, Speer T, Kleber ME (2014). HDL cholesterol is not associated with lower mortality in patients with kidney dysfunction. J Am Soc Nephrol.

[40] Kopecky C, Ebtehaj S, Genser B (2017). HDL cholesterol efflux does not predict cardiovascular risk in hemodialysis patients. J Am Soc Nephrol.

[41] Bauer L, Kern S, Rogacev KS (2017). HDL cholesterol efflux capacity and cardiovascular events in patients with chronic kidney disease. J Am Coll Cardiol.

[42] Wierda WG, O'Brien S, Wang X (2007). Prognostic nomogram and index for overall survival in previously untreated patients with chronic lymphocytic leukemia. Blood..

[43] Xia WK, Liu ZL, Shen D (2016). Prognostic performance of pre-treatment NLR and PLR in patients suffering from osteosarcoma. World J Surg Oncol.

